# Functional microbial shifts and host-microbiome crosstalk in colorectal cancer: insights from a metaproteomic approach

**DOI:** 10.1186/s12866-026-04807-0

**Published:** 2026-02-25

**Authors:** Esraa E. Sobhy, Shahd Ezzeldin, Ahmed Karam, Ahmed Galal, Amany Mokhtar, Wagida Anwar, Amr Abou-Elmagd, Sameh Magdeldin, Shymaa Enany

**Affiliations:** 1 Department of Microbiology and Immunology, Faculty of Pharmacy, Delta University for Science and Technology, Gamasa, 11152 Egypt; 2 Research Department, Proteomics and Metabolomics Research Program, Basic Research Unit, Children’s Cancer Hospital, Cairo, 57357 Egypt; 3 Biomedical Research Department, Armed Forces College of Medicine (AFCM), Cairo, Egypt; 4 Community Medicine Department, Ain Shams University, Cairo, Egypt; 5 Gastroenterology Department, Armed Forces College of Medicine (AFCM), Cairo, Egypt; 6 Physiology Department, Faculty of Veterinary Medicine, Suez Canal University, Ismailia, Egypt; 7 Microbiology and Immunology Department, Faculty of Pharmacy, Suez Canal University, Ismailia, 41522 Egypt

**Keywords:** Colorectal cancer, Metaproteomic, Microbiome, DPP-4, Cysteine

## Abstract

Colorectal cancer (CRC) incidence is increasing in many low- and middle-income countries, including Egypt, partly due to urbanization and lifestyle changes. Metaproteomic approaches remain underutilized in these settings. In this study, we applied fecal metaproteomics in Egyptian CRC patients to characterize disease-associated protein expression patterns, explore host–microbiota functional interactions, and identify metabolic pathways that are altered in the CRC gut environment. Stool samples from 10 CRC patients and 10 healthy controls were analyzed. A total of 441 differentially expressed proteins (DEPs) were identified, of which 406 were consensus proteins overlapping across fold-change analysis, Wilcoxon testing, and PLS-DA. Differential microbial protein expression was primarily associated with core metabolic functions, including carbohydrate, amino acid, and nucleotide transport. Notably, proteins from *Segatella copri* were markedly suppressed in CRC patients. Functional analysis revealed upregulation of microbial proteins related to DPP-4 and cysteine metabolism, suggesting a possible role of microbiome-derived enzymes in colorectal cancer–associated metabolic and immune modulation, without direct evidence of host translocation.

**Supplementary Information:**

The online version contains supplementary material available at 10.1186/s12866-026-04807-0.

## Introduction

 Colorectal cancer (CRC) is a major global health problem, ranking as the third most commonly diagnosed cancer and the second leading cause of cancer-related death globally, with approximately 1.9 million new cases and 935,000 deaths in 2020 alone [1]. It affects both men and women and is extremely prevalent in high-income countries with aging populations and sedentary lifestyles. However, recent trends demonstrate a concerning rise in incidence in individuals under 50 years old [2], and it is expected to rise by the year 2030. CRC predominantly affects older individuals globally, but in Egypt, during the fifth and seventh decades of life, with 25% of the cases occurring in patients under 40 years old [3].

Colorectal cancer incidence and mortality are rising rapidly in most low and middle-income nations, but stabilizing or declining trends are being observed in highly developed countries, which continue to have some of the highest rates in the world [4]. Changes in the epidemiology of colorectal cancer have been observed throughout the Arab world, including Egypt, due to urbanization and changes in lifestyle [5]. CRC is found to be more prevalent in males [6].

The emergence of omics technologies, such as genomics, transcriptomics, proteomics, metabolomics, and metaproteomics, has transformed cancer detection by facilitating thorough molecular profiling of tumors and microenvironments. These high-throughput approaches facilitate the identification of genetic mutations, changes in gene expression, protein signatures, and metabolic alterations associated with cancer initiation and progression. By identifying disease-specific biomarkers and dysregulated pathways, omics systems provide strong tools for early identification, patient stratification, and tailored treatment planning. Metaproteomics, the large-scale characterization of all proteins expressed by microbial communities in a given environment, has emerged as a powerful approach for understanding functional interactions between the gut microbiota and the host in health and disease, particularly CRC [7, 8]. Unlike metagenomics, which reveals microbial gene content, metaproteomics provides direct evidence of active biochemical processes, allowing researchers to link microbial and host protein functions to specific disease phenotypes.

In CRC, the gut microbiota is central to tumor genesis, progression, and immune modulation. However, the precise functional roles of microbial communities are still poorly understood. Metaproteomic investigations offer a functional window to decipher this complexity by revealing microbial enzymes, virulence factors, and host immune-related proteins that change dynamically during carcinogenesis. Current studies have been able to merge host and microbial proteomes to investigate interactions among microbial metabolites and host response in CRC. Because millions of epithelial cells are sloughed into the colon lumen every day, stool-based protein extraction for metaproteomic analysis inevitably consists of both microbial and host-derived proteins [9]. Host secretions such as mucins and immune-related proteins are also present [10]. These host proteins mirror the tissue microenvironment, and feces is an informative and non-invasive source of tissue-specific markers in CRC.

Long et al. (2020) [11] used metaproteomic profiling to identify 341 differential proteins between patients and healthy controls, including 124 proteins upregulated in CRC. *Odoribacter*’s site-specific integrase had the greatest fold change, indicating its significance in DNA binding, integration, and recombination capabilities that may be related to microbial-driven genomic instability in CRC. Iron-regulating proteins, such as bacterioferritin from Parabacteroides, were also upregulated. Long et al. (2020) employed the 20 most discriminatory microbial proteins in a linear support vector machine (LSVM) classifier with an area under the ROC curve of 0.952. These data support metaproteomics as a potent, non-invasive strategy to identify gut-derived microbial biomarkers for early CRC detection.

Tanca et al. (2022) [12] used shotgun metaproteomics to investigate tumor-associated colonic luminal contents, detecting over 30,000 microbial peptides. They discovered that formate-tetrahydrofolate ligase was significantly overexpressed in high-stage and high-grade CRC. Formate produced during anaerobic fermentation by the microbiota or the host can serve as a substrate for both aerobic and anaerobic bacterial growth or enter circulation for nucleotide synthesis [13]. Moreover, they also hypothesized formate competition between CRC and the gut microbiota, implying active microbial metabolic engagement in tumor progression.

Functional profiles can also help guide therapeutic development by revealing microbial pathways that influence chemotherapy efficacy. Bacteria that express β-glucuronidase, such as *Clostridium*,* Eubacterium*,* and Ruminococcus*, can reactivate irinotecan’s toxic metabolite (SN-38) in the gut, leading to severe intestinal side effects [14–16]. Probiotics have been proposed to target this microbial enzyme and prevent irinotecan-induced diarrhea [17]. Similarly, *Fusobacterium nucleatum* increases chemoresistance to 5-fluorouracil by activating immune-related pathways and modifying cancer cell destiny [18].

Despite these developments, metaproteomic research in Egypt is limited. While metagenomics is rapidly being used to treat gastrointestinal and systemic disorders, metaproteomic is underutilized due to issues like difficult sample processing, the necessity for high-resolution mass spectrometry, and limited bioinformatics power. However, interest in functional omics is increasing. This study applied a metaproteomic approach to characterize protein expression profiles in stool samples from Egyptian CRC patients, aiming to explore disease-associated functional patterns, including microbial and host-related pathways linked to host–microbiome interactions, immune modulation, and metabolic alterations.

## Results

### Differential gut microbial proteins between CRC patients and healthy controls

To explore microbial functional differences between CRC patients and healthy controls, we analyzed metaproteomic profiles derived from fecal samples. Across all samples, the identified proteome predominantly reflected microbial proteins, consistent with the use of a customized microbial database. No host-derived proteins were detected, as human sequences were not included in the search database. After preprocessing and quality control, a total of 1,506 proteins were confidently identified. Of these, 532 proteins were found to be differentially expressed, defined by an absolute log₂ fold change (|log₂FC|) ≥ 2.0 (Supplementary Figure S1, which shows significant and non-significant proteins that resulted from fold change analysis). Specifically, 157 proteins were upregulated and 375 were downregulated in CRC patients relative to controls.

**Fig. 1:**
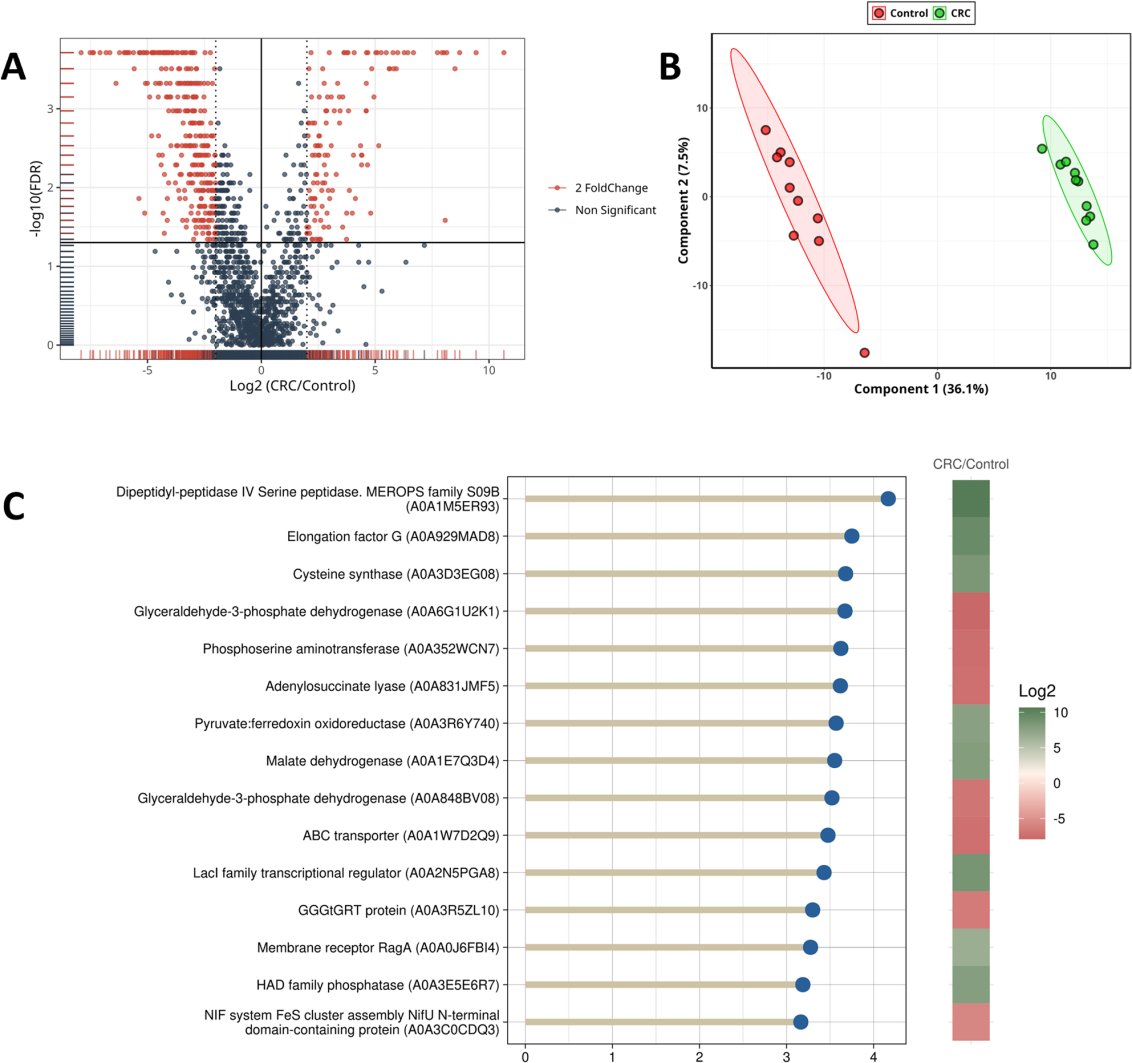
(**A**) Volcano plot shows the integration of fold change analysis with Wilcoxon test to identify significant proteins (red) and non-significant proteins (dark grey). (**B**) A plot showing the results of PLS-DA. (**C**) Top 15 discriminatory proteins from PLS-DA between CRC and control groups. VIP scores (x-axis) indicate each protein’s importance in class separation. color scale represents log₂ fold change (CRC/Control), with green indicating upregulation and red indicating downregulation

Non-parametric statistical analysis using the Wilcoxon test further identified 615 proteins as statistically significant at FDR < 0.05 (Supplementary Figure S2, which shows significant and non-significant proteins that resulted from the Wilcoxon test). Integrating both fold change and statistical significance criteria, 441 proteins met the threshold for both biological and statistical significance (|log₂FC| ≥ 2.0 and FDR < 0.05), as visualized in the volcano plot (Fig. 1). To investigate group separation based on proteomic profiles, hierarchical clustering of the top 200 differentially expressed proteins (DEPs) revealed a clear distinction between CRC and control samples (Supplementary Figure S3, which illustrates distinct clustering of CRC and control samples based on protein expression patterns). This was further supported by principal component analysis (PCA), where PC1 and PC2 accounted for 39.2% and 8.7% of total variance, respectively, showing distinct clustering of CRC and control groups (Supplementary figure S4).

A PLS-DA model was also constructed, which reinforced the separation seen in PCA (Fig. 1). PLS-DA showed an exploratory separation between CRC patients and controls, but permutation testing indicated limited predictive robustness. Model performance was evaluated using cross-validation and permutation testing. The corresponding R² and Q² values are provided in Supplementary Figures S5 and S6. From this analysis, 426 proteins had a VIP score ≥ 1.0, with the top 15 most influential proteins highlighted in Fig. 1. The analysis revealed an exploratory separation between the two groups, with Component 1 accounting for 39.1% and Component 2 explaining 8.2% of the total variance.

**Fig. 2:**
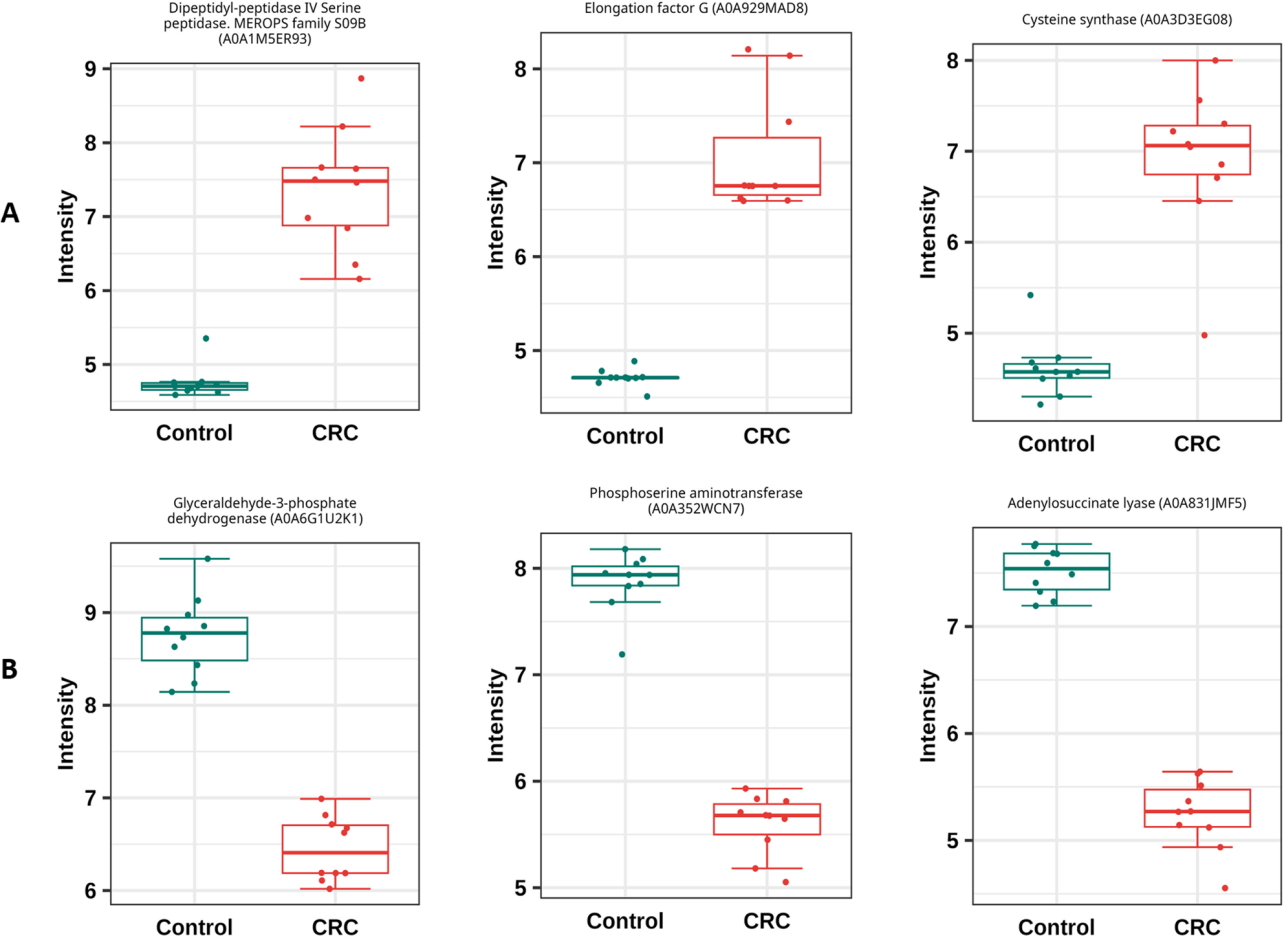
Differences in the number of proteins between CRC and control groups across key COG functional categories. (**A**) represents the top 3 upregulated proteins in CRC, and (**B**) represents the top downregulated proteins in CRC

Among the top 15 discriminatory proteins identified by PLS-DA, eight proteins were upregulated (Fig. 1). Dipeptidyl-peptidase IV serine peptidase (DPP-4) (A0A1M5ER93), a member of the MEROPS family S09B, was identified as the most strongly upregulated in *Bacteroides luti* in CRC patients, as shown in Fig. 2. Elongation factor G (A0A929MAD8) and cysteine synthase (A0A3D3EG08), a key enzyme in sulfur amino acid metabolism [19], were also strongly upregulated in CRC patients, followed by several other proteins such as pyruvate: ferredoxin oxidoreductase (A0A3R6Y740), a central enzyme in anaerobic energy metabolism [20]. Additionally, malate dehydrogenase (A0A1E7Q3D4), a component of the tricarboxylic acid (TCA) cycle and the LacI family transcriptional regulator (A0A2N5PGA8), known for modulating microbial gene expression in response to environmental cues [21], were also upregulated. Other upregulated proteins include the membrane receptor RagA (A0A0J6FBI4), involved in nutrient transport and signaling and HAD family phosphatase (A0A3E5E6R7), which is associated with diverse metabolic regulatory roles [22].

In contrast, among the top 15 discriminatory proteins identified by PLS-DA (Fig. 1), seven proteins were downregulated. These proteins included glyceraldehyde-3-phosphate dehydrogenase (A0A6G1U2K1). It is a central enzyme in glycolysis [23]. Phosphoserine aminotransferase (A0A352WCN7), which is involved in serine biosynthesis [24], and adenylosuccinate lyase (A0A831JMF5) which is an essential enzyme in purine metabolism [25], also showed notable reductions in CRC patients compared to healthy individuals. Other downregulated proteins identified by PLS-DA include glyceraldehyde-3-phosphate dehydrogenase (A0A848BV08), the ABC-type xenobiotic transporter (A0A1W7D2Q9) and GGGtGRT protein (A0A3R5ZL10). The NIF system FeS cluster assembly protein NifU N-terminal domain containing protein (A0A3C0CDQ3) which is essential for the formation of iron-sulfur clusters that support key enzymatic and redox reactions [26], was also downregulated. These observations suggest alterations in metabolic processes linked to the disease state.

## Functional enrichment of differentially expressed proteins

**Fig. 3:**
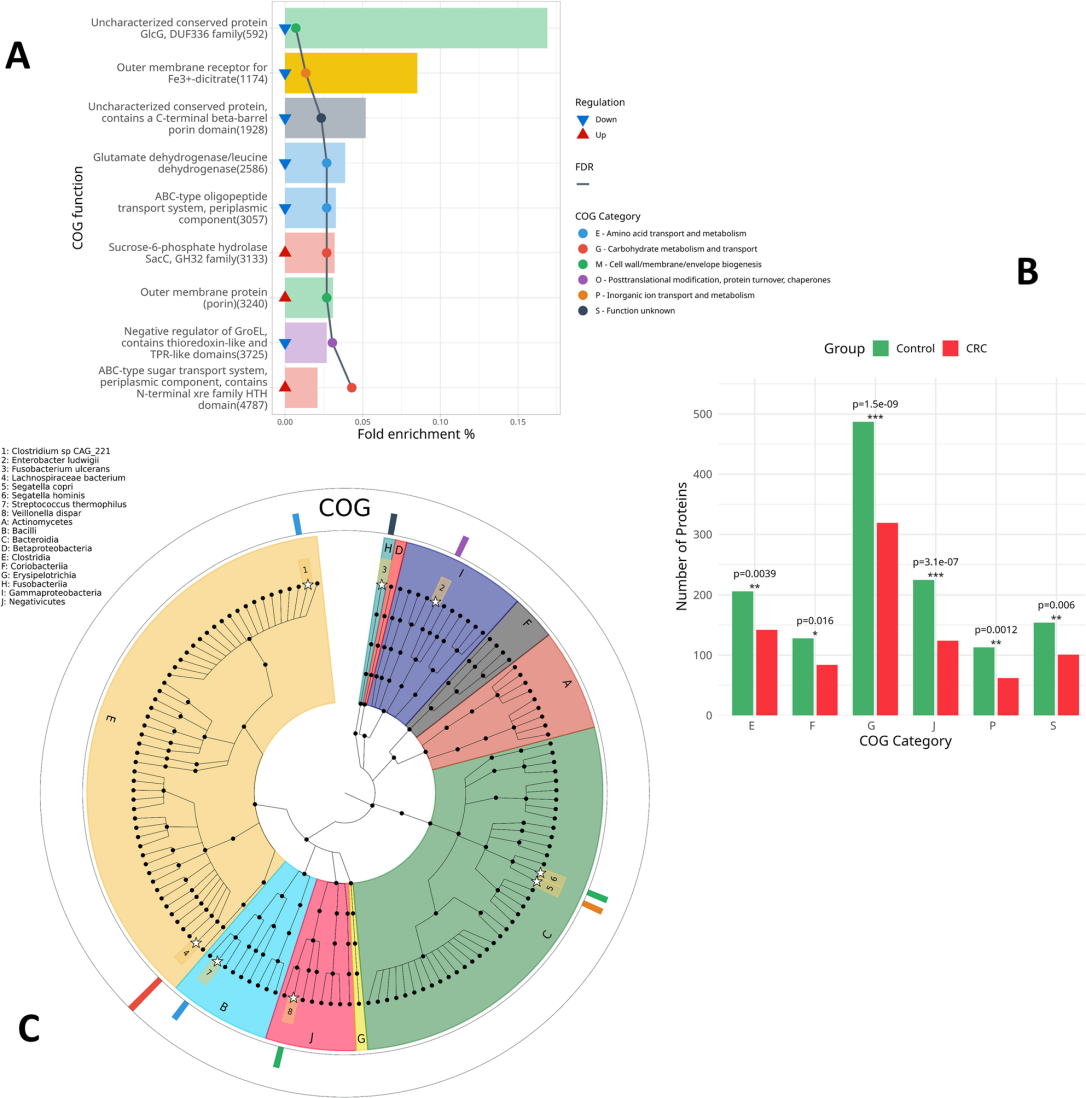
Comparative analysis of microbial protein abundance across functional COG categories in CRC and control groups. (**A**) Barplot shows the COG function of significantly enriched proteins along with their COG category. Fold enrichment % refers to the number of matched proteins with the COG term divided by the total number of proteins in the COG term. (**B**) Bar chart depicting the number of proteins identified in each of six significantly different COG categories between CRC and controls. Statistical significance was determined using Fisher’s exact test, with p-values annotated above each bar pair (*p* < 0.05, *p < 0.01, **p < 0.001). (**C**) Cladogram shows the 135 bacterial species. The 10 inner colors indicate the phyla from which the bacterial species descended (labels from A to J). White stars indicate bacterial species (labels from 1 to 8) that have significantly enriched proteins. The outer ring indicates the COG category of enriched proteins and the number of these proteins in the species

To gain insight into the biological functions of the dysregulated proteins, we intersected the DEPs identified by fold change analysis, Wilcoxon test, and PLS-DA, resulting in 406 consensus proteins. Of these, 374 proteins were successfully annotated by aligning to seed orthologs in the eggNOG 5.0 database. Functional enrichment using the COG database revealed 9 significantly enriched proteins (FDR < 0.05), classified into 6 distinct COG categories (Fig. 3, Supplementary Table S2). Given the limited number of statistically enriched proteins, these findings were interpreted as targeted functional alterations rather than pathway-wide effects.

Six proteins were notably downregulated in CRC patients and significantly enriched in COG. The ABC-type oligopeptide transport system, periplasmic component (R6FSD2), classified under COG4166 (Category E – amino acid transport and metabolism), was significantly downregulated in CRC patients. This protein was taxonomically linked to *Clostridium sp. CAG:221*. This system is known to facilitate oligopeptide uptake [27], and its downregulation may suggest diminished peptide transport activity in CRC-associated microbiota. The uncharacterized protein GlcG, DUF336 family (A0A4Y8VEI4), associated with COG3193 (Category M – cell wall/membrane/envelope biogenesis), was also downregulated and mapped to *Segatella hominis*. GlcG is related to glucose transport [28]. Glutamate dehydrogenase/leucine dehydrogenase (A0A0M4HX97), categorized under COG0334 (Category E) and mapped to *Streptococcus thermophilus*, was downregulated. This enzyme plays a central role in nitrogen metabolism by catalyzing the oxidative deamination of amino acids to the ketons [29]. Similarly, the negative regulator of GroEL (G8LKX5), part of COG3118 (Category O – posttranslational modification, protein turnover, and chaperones), was identified in *Enterobacter ludwigii*. GroEL chaperonin is essential for protein folding under stress [30], and reduced regulation may reflect impaired protein quality control or microbial stress adaptation in CRC associated microbiota. Moreover, the outer membrane receptor for Fe³⁺-dicitrate (A0A3R5Z2W5), linked to *Segatella copri* and classified under COG4772 (Category P – inorganic ion transport and metabolism), was downregulated, suggesting disruption in iron acquisition, a vital process for microbial growth and virulence [31]. Lastly, an uncharacterized conserved protein (A0A2P1S687), containing a C-terminal β-barrel porin domain and affiliated with *Fusobacterium ulcerans*, showed reduced expression. It falls under COG4625 (Category S – function unknown).

Three upregulated proteins were significantly enriched in COG. Sucrose-6-phosphate hydrolase SacC family (A0A646H9P7), belonging to COG1621 (Category G – carbohydrate metabolism and transport), was upregulated in CRC patients and associated with *Lachnospiraceae* species and it is known to participate in levan, inulin, and sucrose degradation [32]. Another upregulated protein, the ABC-type sugar transport system periplasmic protein (A0A929UD98), grouped under COG1879 (Category G), also originated from *Lachnospiraceae*. The concurrent upregulation of these sugar metabolism proteins may indicate enhanced microbial carbohydrate utilization in the tumor microenvironment. Additionally, the outer membrane protein (porin) (A0A6N3CHP0), annotated under COG3203 (Category M – cell wall/membrane/envelope biogenesis), was upregulated in CRC patients and associated with *Veillonella dispar*. Porins are integral membrane proteins that form channels allowing the passive diffusion of small molecules across the outer membrane [33] and their increased abundance may facilitate enhanced nutrient uptake or altered host–microbe interactions in the tumor microenvironment.

A Fisher’s exact test was performed to assess category-level distribution trends and as a complement to the protein-level enrichment results (Fig. 3). This analysis included both proteins unique to CRC or control groups and those shared across both groups. A total of six COG categories showed statistically significant differences in protein abundance between CRC patients and healthy controls (Fisher’s exact test, *p* < 0.05). Notably, the number of proteins related to carbohydrate transport and metabolism (COG G) and translation, ribosomal structure, and biogenesis (COG J) was markedly reduced in CRC patients compared to controls (*p* = 1.5e–09 and 3.1e–07, respectively). Similarly, proteins associated with inorganic ion transport and metabolism (COG P), amino acid transport and metabolism (COG E), and nucleotide transport and metabolism (COG F) were also significantly lower in CRC. These observations indicate selective changes in microbial functional processes rather than global suppression of microbial metabolism.

## Functionally relevant but Non-Enriched proteins reveal microbial shifts in CRC

While only a subset of proteins reached statistical significance in COG enrichment analysis, additional proteins were examined based on consistent differential expression or the established functional relevance in host–microbiome interactions and cancer biology. Among the most consistently downregulated proteins were flagellin subunits (*n* = 14 proteins from the species: *Agathobacter rectalis*,* Bacillus canaveralius*,* Bacillus Bacillus* sp. V33-4, *Clostridium chauvoei*,* Clostridium chauvoei* JF4335, *Clostridium* sp. AM27-31LB, *Clostridium* sp. CAG:122, *Butyribacter intestini*,* Clostridium* sp. CAG:167, *Lachnospira pectinoschiza*,* Pseudomonas* spp., *Roseburia faecis*,* Roseburia intestinalis*,* Roseburia inulinivorans* CAG:15, *Roseburia inulinivorans* DSM 16841, *Selenomonas bovis*). Their reduced abundance is consistent with altered microbial motility or colonization-associated traits. Glyceraldehyde-3-phosphate dehydrogenase (GAPDH) family enzymes (*n* = 9 proteins from species: *Bifidobacterium adolescentis*,* Bifidobacterium faecale*,* Bifidobacteriaceae bacterium* MCC01947, *Bifidobacteriaceae* bacterium MCC01941, *Bifidobacteriaceae* bacterium MCC01943, *Bifidobacterium* sp. KRGSERBCFTRI, *Faecalibacterium prausnitzii*,* Faecalibacterium Faecalibacterium* sp. IP-1–18, *Klebsiella pneumoniae* subsp. pneumoniae NTUH-K2044, *Klebsiella pneumoniae* JM45, *Klebsiella pneumoniae* KP-1, *Klebsiella pneumoniae* 30684/NJST258_2, *Klebsiella pneumoniae* 30660/NJST258_1, *Megasphaera hexanoica*,* Megasphaera* sp. DJF_B143, *Megasphaera*,* Caecibacter massiliensis*,* Prevotella* sp. AM42-24, *Segatella hominis*,* Prevotella* sp. CAG:604, *Prevotella spp.*,* Segatella copri*,* Selenomonas bovis* DSM 23594, *Selenomonas bovis* 8–14-1, *Streptococcus* sp. O’Mahoney, *Streptococcus pseudoporcinus* LQ 940-04, *Streptococcus pseudoporcinus* SPIN 20026, *Streptococcus* sp. NCTC 5968, *Streptococcus* sp. NCTC 10228, *Streptococcus* sp. NCTC 10233, *Streptococcus porcinus*,* Vagococcus entomophilus*). GAPDH is not only important for carbohydrate metabolism but also participates in host–microbe interactions [23].

Also, the reduced expression of cytoplasmic proteins (*n* = 8 proteins from species: *Leyella stercorea* DSM 18206, *Leyella stercorea* CAG:629, *Leyella stercorea*,* Prevotella* sp. AM42-24, *Prevotella* sp. CAG:386, *Segatella hominis*,* Segatella copri* CAG:164, *Segatella copri*,* Prevotellaceae* bacterium, *uncultured Prevotella* sp., *Candidatus Segatella violae*), further support the presence of metabolic remodeling trends within the CRC-associated microbiota. In addition, OmpA family proteins (*n* = 7 proteins from species: *Segatella hominis*,* Prevotella* sp. AM42-24, *Segatella copri*,* Prevotella* sp. AM23-5). OmpA is a key outer membrane protein involved in invasion, host adhesion, structure stability and was downregulated, possibly reflecting weakened bacterial interaction with host tissues [34].

Among downregulated proteins, TonB-dependent receptors, including SusC/RagA family proteins (*n* = 7 proteins; Table 1), which mediate the transport of protein degradation products [22] and carbohydrates [35], were identified. Stress response chaperones (*n* = 5 proteins from species: *Clostridium* sp. CAG:343, *Pseudomonas aeruginosa*,* Roseburia porci*,* Segatella copri)*, which assist in protein folding under harsh conditions [30] and rubrerythrins (*n* = 5 proteins from species: *Clostridium disporicum*,* Clostridium saudiense*,* Clostridium* sp. K04, *Clostridium* sp. AF37-5, uncultured *Clostridium* sp., *Prevotella* sp. TF12-30, *Segatella copri*, uncultured *Prevotella* sp.), which help detoxify reactive oxygen species, were also downregulated, potentially suggesting reduced microbial resilience to oxidative stress [36].

**Table 1:** Differential expression of TonB-dependent receptor proteins related to the SusC/RagA family across various bacterial taxa in CRC patients

Protein ID	Organisms	log2.FC.	*p*.adjusted	Regulation
A0A0J6FBI4	*Parabacteroides goldsteinii*	6.6794	0.000194	Upregulated
A0A840DBA3	*Bacteroides reticulotermitis*	5.8113	0.000311	Upregulated
A0A5B5VLY8	*Alistipes finegoldii*	2.2378	0.045409	Upregulated
A0A3R5YLJ0	*Segatella copri*	−2.5962	0.005168	Downregulated
A0A3R5Z2W5	*Segatella copri*	−3.4472	0.000194	Downregulated
A0A413CBI1	*Segatella copri*	−2.8607	0.000709	Downregulated
A0A5P0WPX8	*Segatella copri*	−2.6116	0.005168	Downregulated
A0A642Q238	*Bacteroides cellulosilyticus*	−4.7171	0.000194	Downregulated
A0A7K0ATR1	*Segatella copri*	−2.88	0.005168	Downregulated
A0A831N7U1	*Phocaeicola vulgatus*	−3.6535	0.000194	Downregulated

In contrast, several protein families were upregulated such as extracellular solute-binding proteins (*n* = 3 proteins from species: *Agathobacter rectalis*,* Bifidobacterium longum* subsp. longum (strain JDM301*)*,* Bifidobacterium longum* subsp. longum CMCC P0001, *Bifidobacterium longum* subsp. suis, *Bifidobacterium longum*,* Bifidobacteriaceae* bacterium MCC01983, *Bifidobacteriaceae* bacterium MCC01979, *Faecalibacterium prausnitzii*). Extracellular solute-binding proteins deliver substrates to the transmembrane transporters like ABC, tripartite ATP-independent periplasmic (TRAP), and tripartite tricarboxylate transporters (TTTs) [37]. Oxidoreductases transferring electrons from pyruvate to flavodoxin (*n* = 3 Proteins from species: *Clostridium* sp. AM33-3, *Clostridium tepidiprofundi* DSM 19306, *Lactococcus insecticola*), which play a key role in anaerobic metabolism [20, 38] were upregulated. Previous studies have reported associations between hypoxic tumor environments and increasing oxidoreductase activity [39]. Finally, peptidases from the M49 family (*n* = 3 Proteins from species: *Bacteroides faecichinchillae*,* Bacteroides intestinalis*,* Segatella copri*) involved in protein degradation are also upregulated, consistent with trends toward enhanced proteolytic potential within CRC-associated microbial communities [40]. 

## Discussion

Overall, the differentially expressed microbial proteins identified in this study converge on three major processes relevant to colorectal cancer: metabolic reprogramming (e.g., altered carbohydrate utilization, anaerobic metabolism, cysteine biosynthesis), immune modulation and evasion (e.g., changes in surface proteins and surface antigen), and suppression of *Segatella copri* proteins. While these associations are observational, they provide mechanistic hypotheses for how microbial functional shifts may accompany CRC development.

Among the most discriminatory proteins identified by PLS-DA, DPP-4 from *Bacteroides luti* (protein ID: A0A1M5ER93) was significantly upregulated among CRC patients. DPP-4 is present in humans and the gut microbiome [41–43]. DPP-4 is long known in human physiology as an enzyme that catalytically inactivates incretin hormones. Incretin hormones, mainly GLP-1 (glucagon-like peptide-1) and GIP (glucose-dependent insulinotropic polypeptide), are secreted from intestinal endocrine cells after a meal. Their primary roles include stimulation of insulin secretion, inhibition of glucagon release, reduction of blood glucose, delay of gastric emptying, preservation of gut mucosal integrity and anti-inflammatory effects [44], thereby modulating glucose metabolism, appetite, and the immune response. Also, human DPP-4 regulates a number of biological processes, including cell differentiation, adhesion, immune modulation and apoptosis, functions that control neoplastic transformation [45].

Interestingly, recent studies have begun to look into its microbial DPP-4 in the human gut. DPP-4-like activity has been detected in symbiotic bacteria such as *Prevotella* and *Lactobacillus*, where it is often associated with membrane-anchored forms of the S9B peptidase family [46, 47]. Using a gnotobiotic mouse model colonized with the normal human microbiota and germ-free mouse, Olivares et al. (2018) offered proof that microbial DPP-4-like activity significantly exceeds activity observed in germ-free controls, suggesting gut bacteria contribute significantly to overall DPP-4 activity in the host. They went on to report that residual plasma DPP-4 activity (~ 10%) observed in DPP-4 knockout mice might arise, at least in part, from microbial sources, with implications for translocation or indirect systemic effects. While direct microbial-to-host transfer of DPP-4 remains unproven, even modest microbial DPP-4 activity may influence host GLP-1 signaling and metabolism. This finding is especially relevant in the case of CRC, where compromised GLP-1 signaling has been linked with cancer development and metabolic imbalance. Specifically, two recent studies reported that increased expression of DPP-4 in CRC tissue correlates with increased metastasis and poorer overall survival [48], while higher circulating levels of DPP-4 have been linked to a greater risk of distant CRC recurrence [45].

**Fig. 4:**
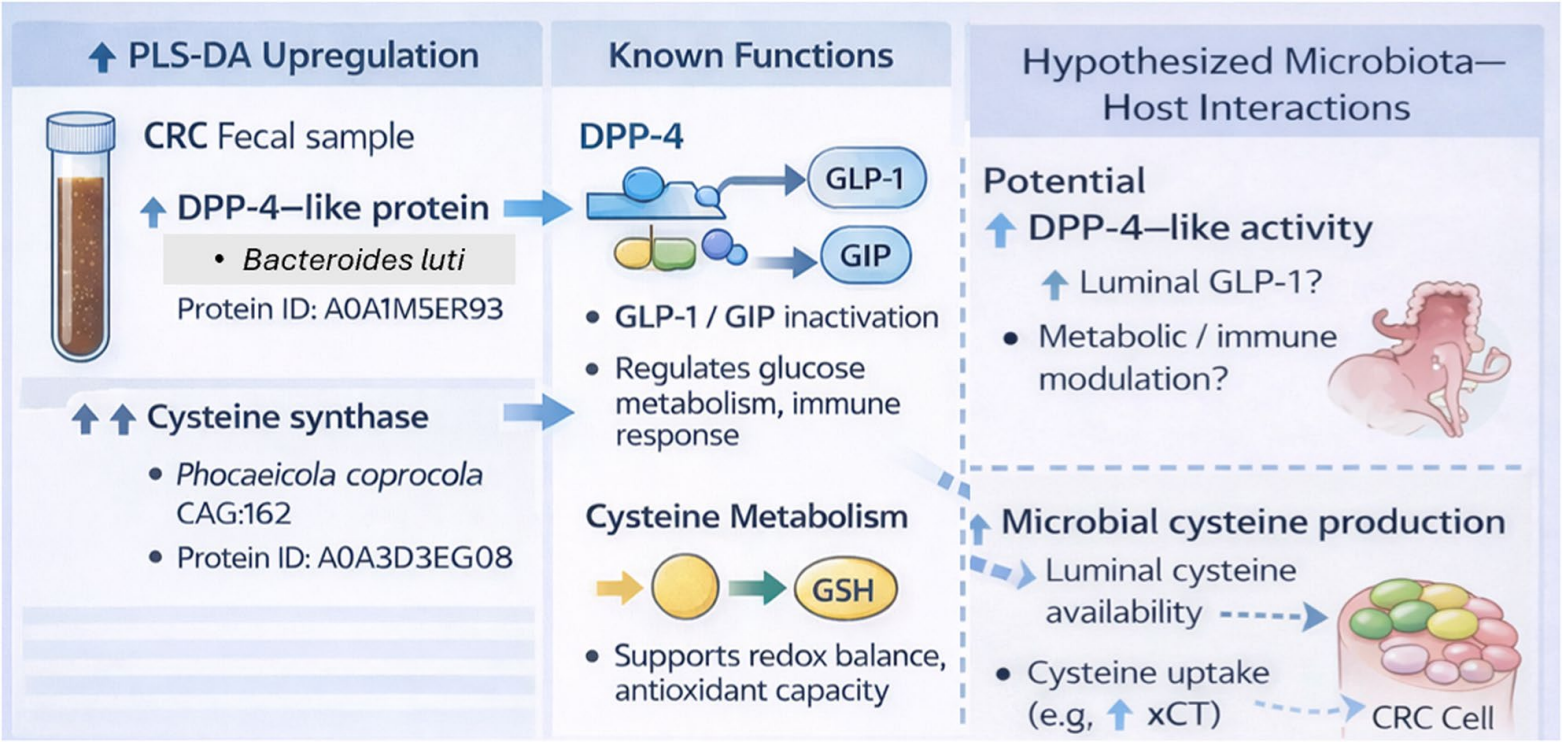
Microbial DPP-4 and cysteine metabolism as potential functional contributors in colorectal cancer

In this study, DPP-4–like proteins were identified exclusively within the fecal metaproteome, reflecting microbial protein expression in the gut lumen. Our findings therefore directly support the presence and differential abundance of microbial DPP-4–like proteins in *Bacteroides luti*. While prior studies have suggested that microbial enzymes may influence host metabolic or immune pathways, our data does not provide direct evidence of microbial DPP-4 translocation across the intestinal barrier or modulation of systemic host DPP-4 activity. Any potential host–microbial interaction remains speculative and should be interpreted as a hypothesis that warrants further investigation using targeted proteomics, host plasma analysis, and functional assays (Fig. 4).

We identified cysteine synthase (protein ID: A0A3D3EG08) among the upregulated microbial proteins in CRC patients, in *Phocaeicola coprocola* CAG:162. The enzyme catalyzes an essential step in the de novo biosynthesis of cysteine, glutathione (GSH) precursor, one of the most critical antioxidants [49]. Gut microbiota-cancer interactions are becoming more important. Cancer patients exhibit elevated levels of body fluid cysteine, which may arise from microbial metabolism, dietary sources, and host pathways such as transsulfuration and protein degradation [50]. CRC cells can also import luminal cysteine via transporters such as xCT, which is more likely to be overexpressed in cancer and associated with oncogenic signaling (e.g., MELK, PI3K, RAS) [51]. Cysteine promotes tumorigenesis and has been proposed as a biomarker for CRC recurrence [52]. Also, studies have shown that cysteine-rich environments facilitate cancer progression. For instance, cysteine-deficient mice had reduced tumor growth and enhanced oxaliplatin sensitivity [51]. The interaction between microbiota and cancer cells in the context of cysteine is focused on two primary mechanisms [50]: (1) cysteine produced by gut microbes can directly serve as a source of energy for the metabolic requirements of cancer cells, and (2) microbial metabolites of cysteine, such as glutathione and hydrogen sulfide (H₂S), can support cancer cell growth by acting as antioxidants and regulating metabolic and energy pathways of cancer cells.

Pyruvate: ferredoxin oxidoreductase (PFOR) (protein ID: A0A3R6Y740) was one of the most discriminatory proteins under PLS-DA analysis. It was derived from *Clostridium sp.* AM33-3. Upregulation refers to a general increase in anaerobic energy metabolism characteristic of microbial adaptation to the hypoxic tumor environment. Notably, Wang et al. (2022) [53] demonstrated that PFOR is an IgA-binding antigen of *Faecalibacterium prausnitzii*. The group confirmed its immunoreactivity with PCR, ELISpot assays, and single-cell RNA sequencing of PBMCs, which hinted that PFOR may be a microbial immunologically active protein involved in host–microbiome interactions.

According to the extensive knowledge of TonB-dependent receptor SusC/RagA family across Bacteroidetes. It was hypothesised that cell surface-associated RagA in *P. gingivalis* was involved in the translocation of protein degradation products, unlike SusC which is involved in a saccharide transport function [22]. We observed that TonB-dependent receptor SusC/RagA family showed contrasting expression patterns across different bacterial taxa (Table 1). This variation likely reflects species-specific adaptations to the CRC gut environment. While some bacteria may reduce their reliance on TonB-dependent transport systems, others may increase their expression to support enhanced nutrient uptake or survival under altered conditions. These findings point to the dynamic and diverse ways in which gut microbes respond to the metabolic pressures of the tumor microenvironment.

In our data, OmpA family proteins (7 proteins) from organisms like *Segatella hominis*, *Prevotella* sp. AM42-24, *Segatella copri*, and *Prevotella* sp. AM23-5 were downregulated in CRC patients. OmpA proteins are outer membrane multifunctional proteins that have crucial functions in bacterial pathogenicity, including structural stabilization, host cell adhesion, serum resistance, and immune modulation [54]. For example, OmpA was reported in *Bacteroides fragilis* to stimulate the production of pro-inflammatory cytokines like IL-1α, TNF-α, IFN-γ, IL-6, and IL-10 from murine immune cells with the possible ability to trigger immune activation [55]. On the other hand, there are bacteria which might use reduced OmpA expression as a means of decreasing host immune detection. Toward this end, *Klebsiella pneumoniae* was found to manipulate the expression of OmpA in order to inhibit the activation of airway epithelial cells and block cytokine responses such as TNF-α and IL-6, thereby enhancing bacterial survival in the host [56]. These findings suggest that the downregulation of OmpA observed in our CRC-associated bacteria is a microbial strategy for immune evasion in the tumor microenvironment, where persistent inflammation and altered host responses would predispose bacteria with lower immunogenic surface expression.

GAPDH, equivalent to protein A0A6G1U2K1 from *Segatella copri*, was highly downregulated and the most discriminative downregulated protein discovered through PLS-DA analysis. Besides *Segatella copri*, Nine GAPDH proteins from species of the genera *Bifidobacterium*, *Faecalibacterium*, *Klebsiella*, *Megasphaera*, *Caecibacter*, *Prevotella*, *Selenomonas*, *Streptococcus*, and *Vagococcus* were also shown to be regularly downregulated in our research. These microorganisms are routinely obligate or facultative anaerobes. GAPDH (EC 1.2.1.12) is a universally expressed enzyme that plays an essential function in glycolysis by the generation of 1,3-bisphosphoglycerate [23]. Because it’s a housekeeping protein, it is essential to the minimal metabolism of almost all organisms, including those with incomplete tricarboxylic acid cycles. In such cases, GAPDH is primarily responsible for maintaining anaerobic or oxygen-limited growth [23]. In *Streptococcus pyogenes*, GAPDH is also surface-located, where it can bind to extracellular matrix components such as fibronectin and laminin, facilitating colonization and survival within the host gut [57]. The enzyme further facilitates the binding of group A *Streptococcus* and closely related streptococci to human plasminogen, enhancing their adhesion to pharyngeal epithelial cells [58]. Studies in *S. pyogenes* and *E. coli* have proceeded to suggest that GAPDH from bacteria could be engaged in non-metabolic processes, such as regulation of transcription, DNA repair assistance, and participation in quorum sensing [59–61].The observed downregulation of GAPDH in *Segatella copri* and other genera in CRC might thus reflect an adaptive strategy to minimize immune recognition by reducing surface-exposed immunogenic factors, particularly in an inflamed or immune-surveilled tumor microenvironment.

Our findings demonstrated an extreme downregulation of various proteins from *Segatella copri* in CRC patients, including GAPDH, rubrerythrins, stress response chaperones, outer membrane proteins such as OmpA, and TonB-dependent receptor proteins related to the SusC/RagA family. The consistent suppression of multiple protein families derived from *Segatella copri* suggest, but do not directly demonstrate, adaptive responses of distinct microbial taxa to the altered nutrient availability, hypoxic conditions, and immune pressures characteristic of the colorectal tumor microenvironment, rather than redundant observations. In contrast, Sepúlveda-Pontigo et al. (2025) [62] reported increased abundance of *Segatella copri* in inflammatory bowel diseases such as rheumatoid arthritis (RA), while Xiao et al. (2024) [63] indicated its involvement in host gene expression regulation, such as the regulation of long non-coding RNAs (lncRNAs) that have been involved in gastric and colorectal cancer in mouse models.

Remarkably, the periplasmic subunit of the ABC-type oligopeptide transport system (R6FSD2), showed downregulation in CRC patients. On the other hand, the ABC-type sugar transport system periplasmic protein (A0A929UD98) was upregulated in CRC and highly enriched in COG. This finding contrasts with those of Buetas et al. (2025) [64], who conducted a metatranscriptomic study of tumor-associated microbiota of colon cancer patients treated at a Spanish hospital. The study of Buetas et al. (2025) revealed downregulation of ABC glucose transporters and upregulation of oligonucleotide transporters in cancer patients.

A key limitation of this study is the relatively small sample size (10 CRC patients and 10 healthy controls) which limits statistical power and reduces the generalizability of the findings. PLS-DA in this study was used as an exploratory tool to highlight discriminatory patterns rather than to establish predictive performance. The supervised nature of PLS-DA, combined with the small cohort size, increases the risk of overfitting, as reflected by non-significant permutation testing results. Consequently, PLS-DA findings should be interpreted as hypothesis-generating rather than confirmatory. Accordingly, proteins identified as discriminatory or biologically relevant should be considered preliminary candidates, and validation in larger, independent cohorts will be required before any diagnostic or prognostic relevance can be inferred. All participants in this study were male, which represents a limitation. Sex-related differences in the gut microbiome have been reported, and the findings may therefore not be directly generalizable to females.

## Conclusion

In summary, this study provides metaproteomic insights into the gut microbiota of Egyptian colorectal cancer patients, revealing differential microbial protein expression linked to metabolic reprogramming, immune modulation, and microbial adaptation to the tumor microenvironment. Among the most notable observations was the increased abundance of microbial DPP-4–like proteins in the fecal metaproteome of CRC patients. While these proteins were detected exclusively at the microbial level, their differential expression suggests a potential role in host–microbiome metabolic or immune interactions previously described in the literature. However, their utility as biomarkers or mediators of systemic effects remains preliminary and requires validation in larger cohorts and targeted functional studies. Also, Further studies with integrated multi-omics strategies are necessary to validate these observations and identify therapeutic targets in the microbiome-host axis.

## Materials and methods

### Participants

This study involved 20 participants, equally divided into two groups: CRC patients (*n* = 10, all male) and healthy male controls (*n* = 10). The groups were matched for age and gender, with mean ages of 53.9 ± 4.8 years in the CRC group and 53.5 ± 3.7 years in the control group. Fecal samples were obtained from individuals undergoing colonoscopy and histopathological evaluation at Kobri Elkoba Hospital in Cairo, Egypt. CRC patients were classified according to the Union for International Cancer Control (UICC) Tumor-Node-Metastasis (TNM) staging system [65]. Individuals were excluded from the study if they had received radiation, chemotherapy, antibiotics, NSAIDs, statins, or probiotics within two months before enrollment, or if they had any confirmed condition other than CRC. Healthy controls demonstrated normal results in blood tests, endoscopy, and/or diagnostic imaging, with no prior disease history or known genetic abnormalities. Demographic and clinical characteristics of study participants, including age, gender, body mass index, smoking status, exercise habits, and tumor-related parameters, are summarized in Supplementary Table S1.

## Samples collection

Fresh stool samples were obtained before any surgical procedure or bowel preparation. All samples were collected promptly and immediately stored at − 80 °C until further processing.

### Protein extraction and in-Gel digestion

Proteins were recovered from fecal samples using a modified filtration process, as previously described [66]. To remove debris, 1 g of fecal sample was rinsed in cold PBS, homogenized for 15 min, then centrifuged at 300 × g at 4 °C for 5 min. The supernatant was collected, and the pellet was resuspended in 2 mL of PBS and vortexed. After washing, the supernatants were pooled and precipitated with acetone at −20 °C overnight. The precipitated proteins were recovered by centrifugation at 12,000 × g at 4 °C for 30 min and dissolved in lysis buffer (4% SDS in 100 mM Tris-HCl, pH 8.5). To decrease disulfide bonds, use 2 µL of 1 M Tris(2-carboxyethyl) phosphine (TCEP). To suppress proteolysis, 100 µL of cOmplete™ Mini EDTA-free Protease Inhibitor Cocktail (Roche, Mannheim) was used. After a second acetone precipitation, the lysate was dissolved in 8 M urea in 500 mM Tris-HCl (pH 8.5), and protein content was measured at 562 nm using the Pierce bicinchoninic acid (BCA) assay (Pierce, Rockford, IL) before digestion. SDS-polyacrylamide gel electrophoresis (SDS-PAGE) was used to separate the samples, and each lane was divided into five equal fractions (a total of 100 fractions) for in-gel tryptic digestion, as described previously [66].

### Metaproteomics profiling using DDA-based LC-MS/MS

Peptide samples were analyzed using liquid chromatography-tandem mass spectrometry (LC-MS/MS) on an EASY-nanoLC 1200 system coupled to an Orbitrap Fusion Lumos Tribrid mass spectrometer (Thermo Fisher Scientific, USA) equipped with a NanoFlex ion source. Approximately 100 ng of peptides were first loaded onto a Thermo Scientific™ Acclaim™ PepMap™ 100 trap column (75 μm inner diameter, 2 cm length, 3 μm C18 particles) and subsequently separated on a 25 cm analytical column (75 μm i.d., 2 μm C18 particles). Peptide elution was performed using a 60-minute gradient of solvent B (0.1% formic acid in 80% acetonitrile), progressing as follows: 5–30% from 0 to 31 min, 30–40% from 31 to 41 min, and 40–80% from 41 to 51 min. This was held for 4 min before ramping to 100% over 5 min at a constant flow rate of 250 nL/min [67].

Mass spectrometric analysis was performed in data-dependent acquisition (DDA) mode with a cycle time of 3 s. Full MS scans were acquired over a mass range of m/z 400–1800 at a resolution of 120,000, with the automatic gain control (AGC) target and maximum injection time set to auto. Monoisotopic precursor selection was enabled, and precursor ions with charge states between 2 and 7 and intensities exceeding 5 × 10³ were selected for fragmentation. Isotopic peaks were excluded, and dynamic exclusion was applied for 30 s with a 10 ppm mass tolerance. MS/MS scans were conducted using higher-energy collisional dissociation (HCD) at a normalized collision energy of 30%, with a quadrupole isolation window of 1.5 m/z [67].

### Metaproteomics data preprocessing

Peptide and protein identification was performed using Proteome Discoverer software (version 2.4.0.305, Thermo Scientific) with the Sequest HT search engine. Spectra were searched against a customized microbial protein database derived from UniProt, comprising 21,949,895 bacterial protein entries. This database was designed to specifically capture microbial protein diversity, thereby ensuring that identified proteins originated exclusively from microbial sources.

The search parameters permitted up to two missed cleavages by trypsin and required a minimum peptide length of six amino acids. Mass tolerances were set at 20 ppm for precursor ions and 0.5 Da for fragment ions. Carbamidomethylation of cysteine residues (+ 57.02146 Da) was defined as a fixed modification, while variable modifications included methionine oxidation (+ 15.995 Da), N-terminal and lysine acetylation (+ 42.01 Da), and the formation of pyrrolidone from carbamidomethylated cysteine (− 17.03 Da).

The Minora Feature Detector was used to compare CRC patients with healthy controls utilizing label-free quantification. Peptide and protein data were filtered and only those entries with a low FDR were included to ensure reliability. For additional examination, only peptides that were at least seven amino acids long were taken into consideration for further analysis [68]. To account for variations in sample dilution, probability quotient normalization (PQN) was applied. Proteins with more than 30% missing values within a group were excluded from the dataset. For the remaining missing values, imputation was performed by inserting random numbers within ± 1.0% of the median for each group. The data were then log₁₀-transformed, and the distribution of values was evaluated for normality using the Shapiro–Wilk test.

### Metaproteomics statistical data analysis

To identify differentially expressed proteins (DEPs) between the two independent groups, fold change analysis was conducted using a threshold of 2.0. The Wilcoxon Mann–Whitney test was applied, and proteins with FDR-adjusted p-value of < 0.05 were considered statistically significant [69]. Hierarchical clustering was then carried out based on the identified DEPs, using Euclidean distance to assess similarity and Ward’s method as the clustering algorithm. To further explore patterns in the data and the sources of variation between CRC patients and healthy controls, PCA and partial least squares discriminant analysis (PLS-DA) were employed.

### Metaproteomics enrichment analysis

To identify proteins for enrichment analysis, the same differentially expressed protein set was used, defined by |log₂FC| ≥ 2.0, a false discovery rate (FDR) < 0.05, and VIP ≥ 1.0. PLS-DA model robustness was assessed using cross-validation and permutation testing. Given the limited sample size, PLS-DA was used for exploratory purposes only and not as a predictive model. Protein identities and sequences were retrieved from the UniParc database. Data analysis was carried out using a combination of custom in-house scripts, along with the MetaboAnalystR and ggplot2 packages [70, 71]. Protein sequences were aligned to the eggNOG 5 database using EggNOG-mapper v2 to identify seed orthologs, applying thresholds of ≥ 50% for identity, query coverage, and subject coverage [72, 73]. Functional enrichment was conducted in iMetaLab using seed ortholog IDs, with Clusters of Orthologous Groups (COG) as the selected enrichment category and a significance threshold of FDR < 0.05 [74, 75]. Taxonomic lineage information for each species was retrieved through iMetaLab’s Phylo Explorer module [75]. To assess differences in category enrichment between groups, Fisher’s exact test was applied to each COG category using 2 × 2 contingency tables. Resulting p-values were adjusted using the Benjamini–Hochberg FDR method, and categories with adjusted p-values < 0.05 were considered significant.

## Supplementary Information

Supplementary Material 1.

## Data Availability

The mass spectrometry proteomics data have been deposited to the ProteomeXchange Consortium via the PRIDE partner repository with the dataset identifier PXD067235 (https:/www.ebi.ac.uk/pride/archive/projects/PXD067235).

## Abbreviations

AGC: Automatic Gain Control
ATP: Adenosine Triphosphate
BCA: Bicinchoninic Acid
COG: Clusters of Orthologous Groups
CRC: Colorectal Cancer
DDA: Data-dependent Acquisition
DEP: Differentially Expressed Proteins
DPP-4: Dipeptidyl peptidase-4
FDR: False Discovery Rate
GAPDH: Glyceraldehyde-3-phosphate Dehydrogenase
GIP: Glucose-dependent Insulinotropic Polypeptide
GLP-1: Glucagon-like Peptide-1
GSH: Glutathione
H₂S: Hydrogen Sulfide
HCD: Higher-energy Collisional Dissociation
LC-MS/MS: Liquid Chromatography-tandem Mass Spectrometry
lncRNA: Long Non-coding RNA
PCA: Principal Component Analysis
PFOR: Pyruvate Ferredoxin Oxidoreductase
PLS-DA: Partial Least Squares Discriminant Analysis
PQN: Probability Quotient Normalization
RA: Rheumatoid Arthritis
SDS-PAGE: Sodium dodecyl sulfate-polyacrylamide gel electrophoresis
TCA: Tricarboxylic Acid
TCEP: Tris(2-carboxyethyl)phosphine
TNM: Tumor-Node-Metastasis
TRAP: ATP-independent Periplasmic Transporter
TTT: Tripartite Tricarboxylate Transporter
UICC: Union for International Cancer Control
VIP: Variable Importance in Projection
