# Supplementary material for: Functional microbial shifts and host-microbiome crosstalk in colorectal cancer: insights from a metaproteomic approach
Source: BMC Microbiol. 2026 Feb 25;26:294. doi: 10.1186/s12866-026-04807-0 (PMC13041051; doi:10.1186/s12866-026-04807-0)
Supplement: Supplementary file 1 — Supplementary Material 1. [file 12866_2026_4807_MOESM1_ESM.docx]

**Supplementary Tables**

**Supplementary Table S1: the clinical and pathological characteristics of the participants.**

| **Group** | **ID** | **Age** | **Gender** | **Body mass index, kg/m2** | **Smoking** | **Exercise** | **CEA ng/ml** | **Disease stage** | **Tumor location** | **Degree of Tumor Differentiation** | **Lymph node metastasis** | **Distant metastasis** |
| --- | --- | --- | --- | --- | --- | --- | --- | --- | --- | --- | --- | --- |
| CRC | P1 | 44 | Male | 24.6 | No | Yes | 6.1 | StageⅠ | Ascending | Low | No | - |
|  | P2 | 54 |  | 39.5 | Yes | No | 24.1 | Stage Ⅲ | Transverse | High | Yes | No |
|  | P3 | 53 |  | 30.2 | Yes | Yes | 11.1 | Stage Ⅰ | Sigmoid | Low | No | - |
|  | P4 | 56 |  | 35.1 | Yes | No | 9.4 | Stage Ⅱ | Ascending | Low | Yes | No |
|  | P5 | 54 |  | 28.5 | Yes | No | 27.1 | Stage Ⅲ | Ascending | High | Yes | Yes |
|  | P6 | 51 |  | 32.2 | Yes | Yes | 19.3 | Stage Ⅳ | Sigmoid | High | Yes | Yes |
|  | P7 | 60 |  | 31.8 | Yes | No | 30.8 | Stage Ⅳ | Rectum | High | Yes | Yes |
|  | P8 | 58 |  | 24.3 | Yes | No | 22.3 | Stage Ⅳ | Rectum | High | Yes | Yes |
|  | P9 | 59 |  | 25.6 | No | No | 15.9 | Stage Ⅲ | Rectum | High | Yes | No |
|  | P10 | 50 |  | 27.9 | No | No | 18.9 | Stage Ⅲ | Sigmoid | High | Yes | No |
| Control | C1 | 52 |  | 25.3 | No | Yes | NA | NA | NA | NA | **NA** | NA |
|  | C2 | 48 |  | 27.2 | No | Yes |  |  |  |  |  |  |
|  | C3 | 49 |  | 26.3 | No | Yes |  |  |  |  |  |  |
|  | C4 | 59 |  | 22.1 | No | Yes |  |  |  |  |  |  |
|  | C5 | 57 |  | 25.7 | No | No |  |  |  |  |  |  |
|  | C6 | 54 |  | 26.3 | No | No |  |  |  |  |  |  |
|  | C7 | 51 |  | 24.3 | No | No |  |  |  |  |  |  |
|  | C8 | 52 |  | 26.9 | No | No |  |  |  |  |  |  |
|  | C9 | 56 |  | 23.5 | No | No |  |  |  |  |  |  |
|  | C10 | 57 |  | 32.3 | Yes | No |  |  |  |  |  |  |

**Supplementary Table S2. Significantly Differentially Expressed Proteins (FDR ≤ 0.05) Categorized by COG Functional Classes**

| **Protein name** | **Protein function** | **COG ID** | **COG Category** | ***Species*** | **Log2(FC)** | **FDR** | **Regulation** |
| --- | --- | --- | --- | --- | --- | --- | --- |
| **R6FSD2** | ABC-type oligopeptide transport system, periplasmic component(3057) | COG4166 | E - Amino acid transport and metabolism | *Clostridium sp. CAG:221* | -3.0384 | 0.00124230 | Downregulated |
| **A0A0M4HX97** | Glutamate dehydrogenase/leucine dehydrogenase(2586) | COG0334 | E - Amino acid transport and metabolism | *Streptococcus thermophilus* | -2.7012 | 0.010896 | Downregulated |
| **A0A646H9P7** | Sucrose-6-phosphate hydrolase SacC, GH32 family(3133) | COG1621 | G -Carbohydrate metabolism and transport | *Lachnospiraceae bacterium* | 4.3593 | 0.00242490 | Upregulated |
| **A0A929UD98** | ABC-type sugar transport system, periplasmic component, contains N-terminal xre family HTH domain(4787) | COG1879 | G - Carbohydrate metabolism and transport | *Lachnospiraceae bacterium* | 3.9947 | 0.021157 | Upregulated |
| **A0A4Y8VEI4** | Uncharacterized conserved protein GlcG, DUF336 family(592) | COG3193 | M - Cell wall/membrane/envelope biogenesis | *Segatella hominis* | -2.7163 | 0.00315920 | Downregulated |
| **A0A6N3CHP0** | Outer membrane protein (porin)(3240) | COG3203 | M - Cell wall/membrane/envelope biogenesis | *Veillonella dispar* | 2.3109 | 0.017351 | Upregulated |
| **G8LKX5** | Negative regulator of GroEL, contains thioredoxin-like and TPR-like domains(3725) | COG3118 | O - Posttranslational modification, protein turnover, chaperones | *Enterobacter ludwigii* | -2.9849 | 0.00035664 | Downregulated |
| **A0A3R5Z2W5** | Outer membrane receptor for Fe3+-dicitrate(1174) | COG4772 | P - Inorganic ion transport and metabolism | *Segatella copri* | -3.4472 | 0.000194 | Downregulation |
| **A0A2P1S687** | Uncharacterized conserved protein, contains a C-terminal beta-barrel porin domain (1928) | COG4625 | S - Function unknown | *Fusobacterium ulcerans* | -1.9835 | 0.031826 | Downregulated |

**Supplementary figures**


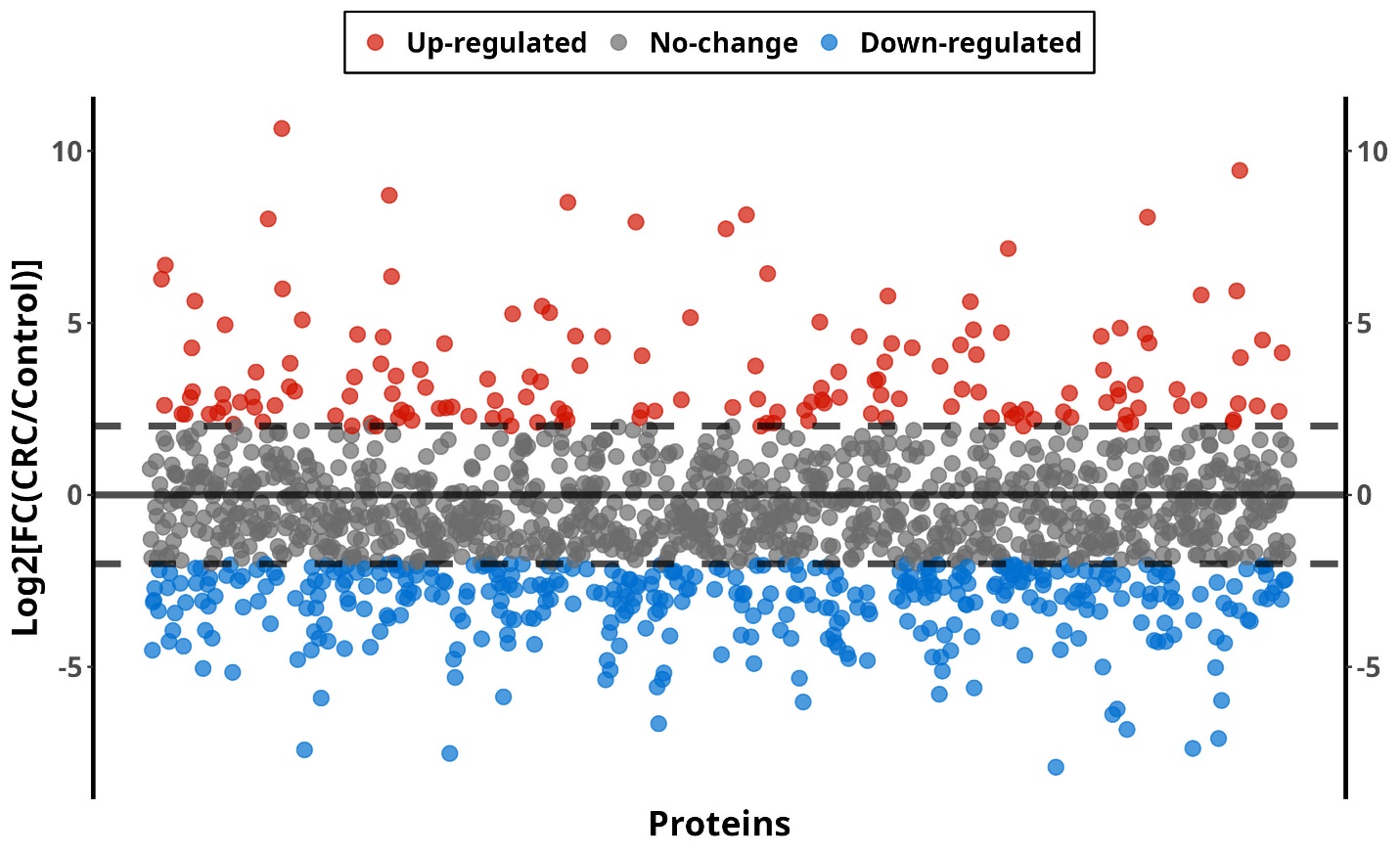


**Supplementary Figure S1**: Dot plot showing significant and non-significant proteins that resulted from fold change analysis. Dashed lines indicate log2 fold change of -2.0 and 2.0.


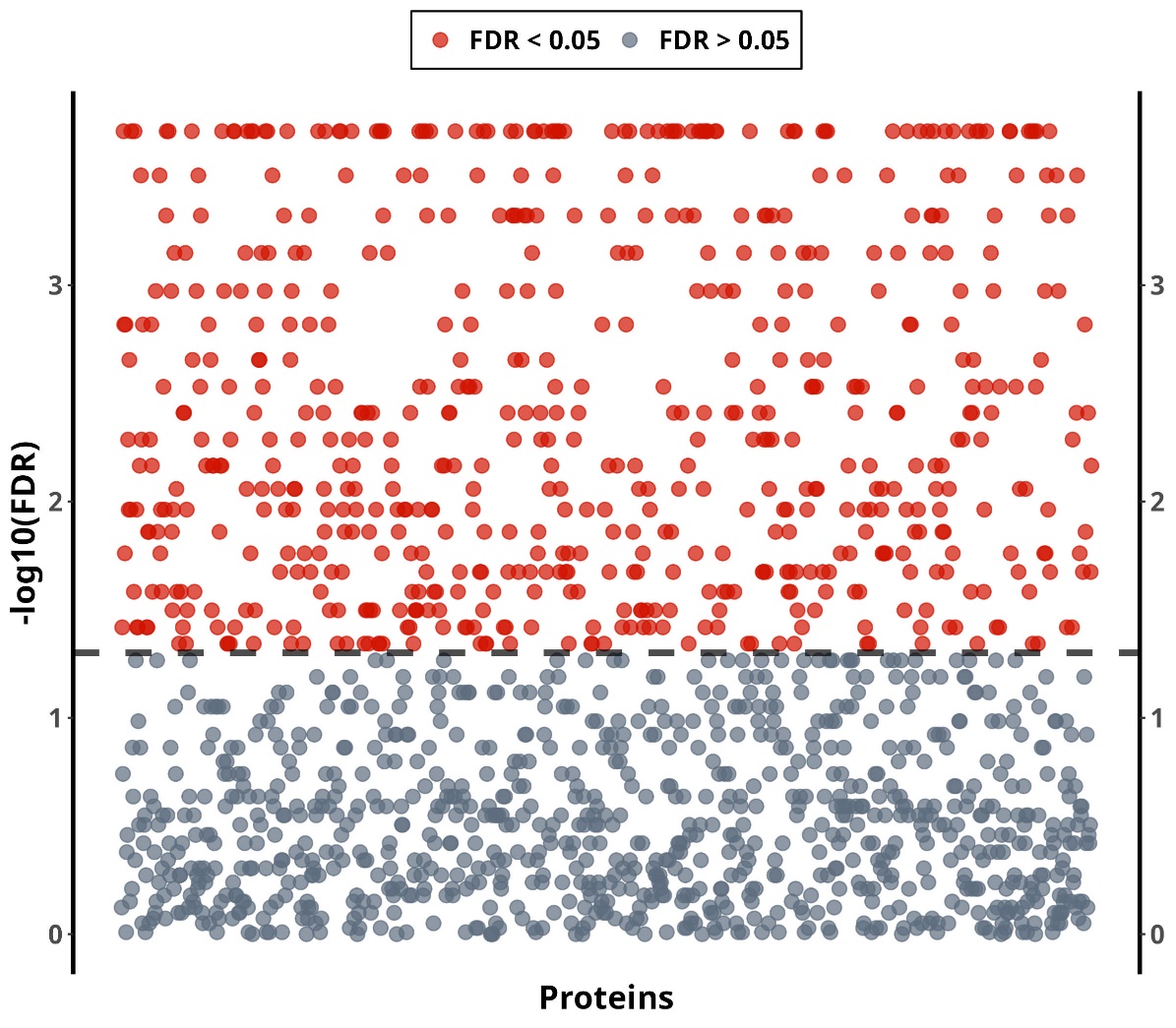


**Supplementary Figure S2**: Dot plot showing significant and non-significant proteins that resulted from the Wilcoxon test. The dashed line indicates FDR = 0.05.


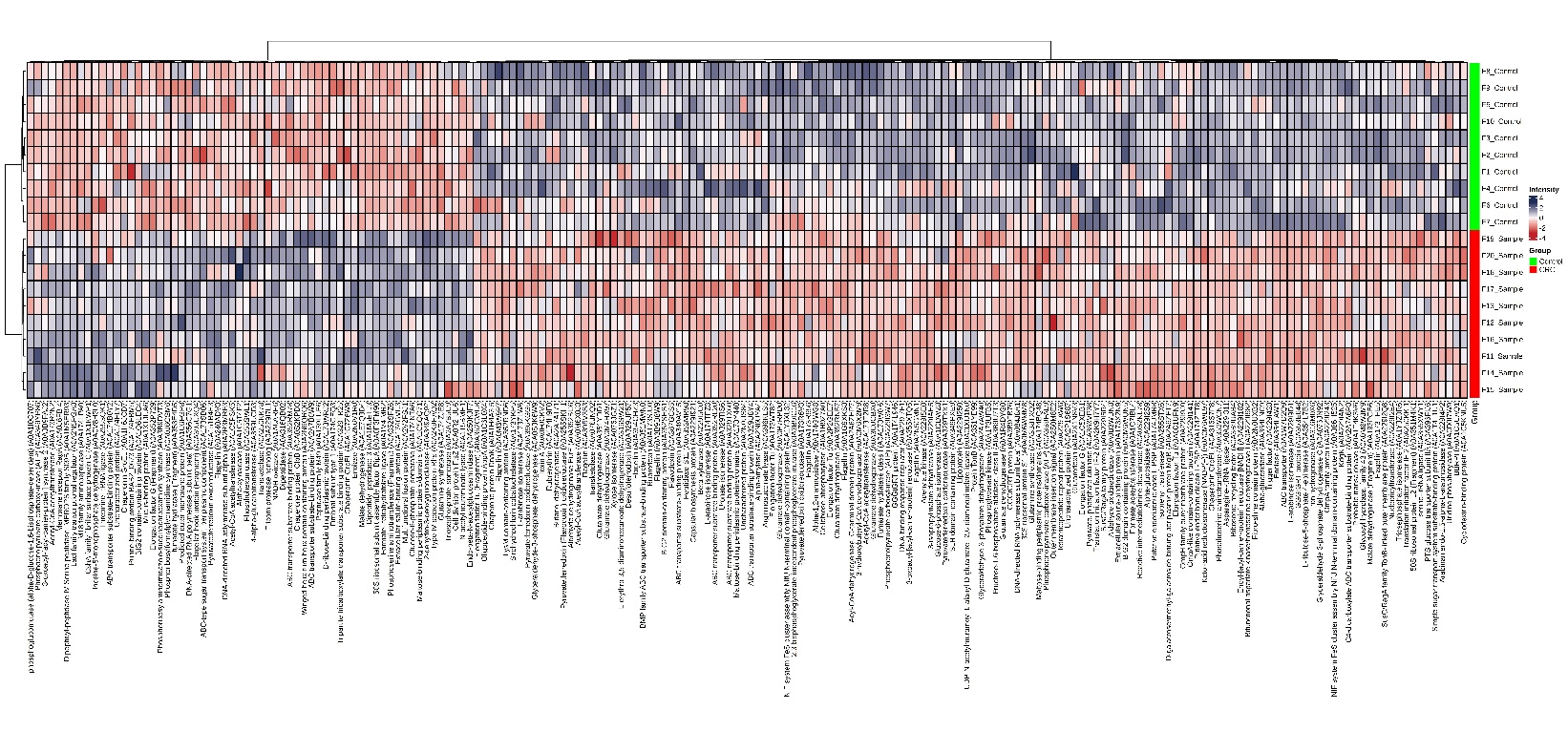


**Supplementary Figure S3**: The heatmap shows the top 200 significant proteins resulting from volcano analysis on the horizontal axis and the CRC and control groups on the vertical axis. The result of the group clustering is that the proteins clearly separate the two groups.


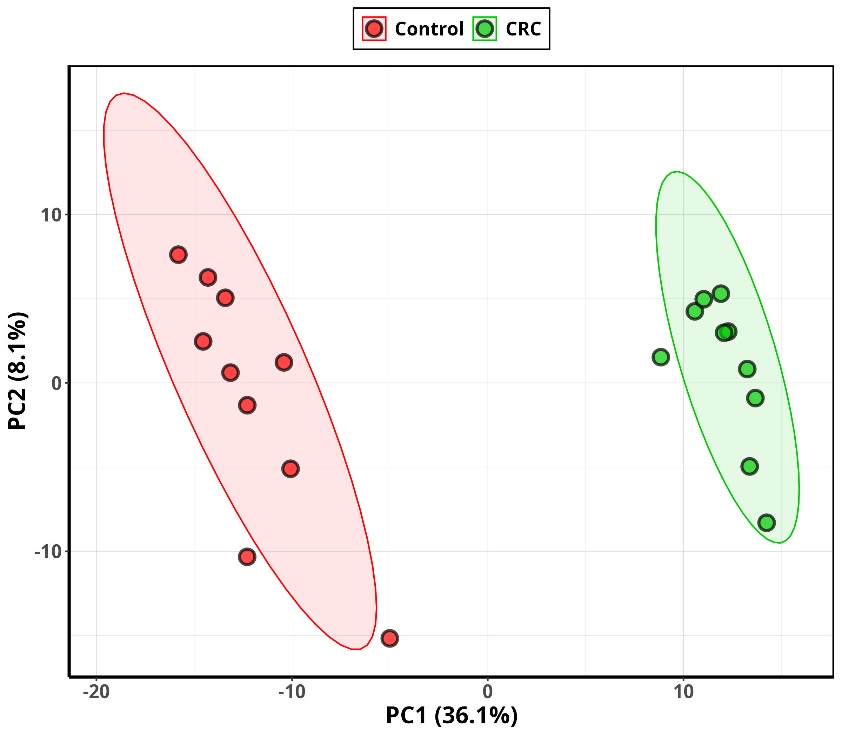


**Supplementary Figure S4:** Dot plot showing the results of PCA. The differences in the protein level between the two groups were sufficient to separate the two groups without supervision.


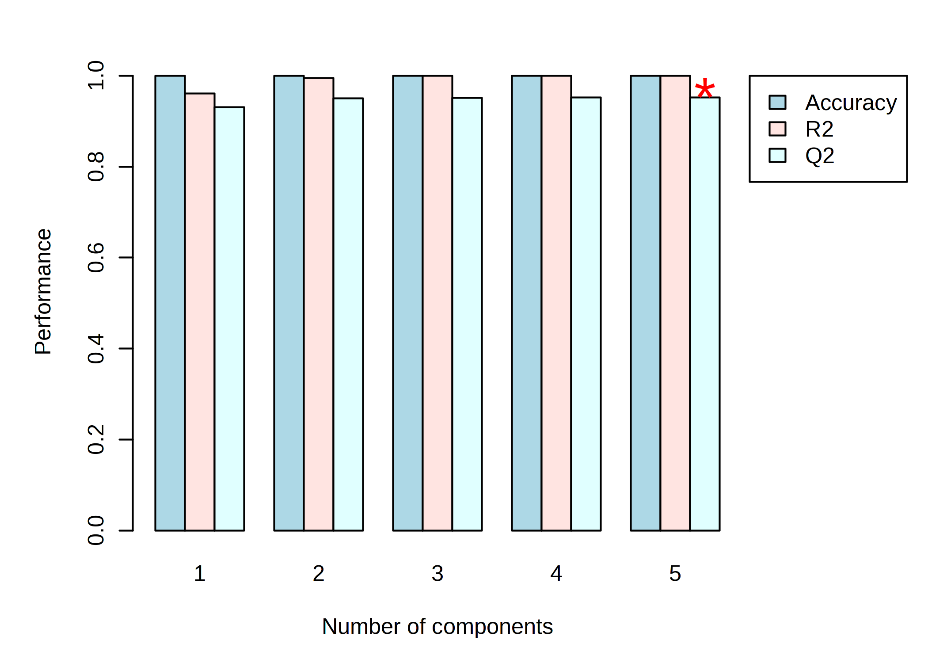


**Supplementary Figure S5:** PLS-DA model validation, cross-validated model performance (accuracy, R², Q²) across increasing numbers of components.


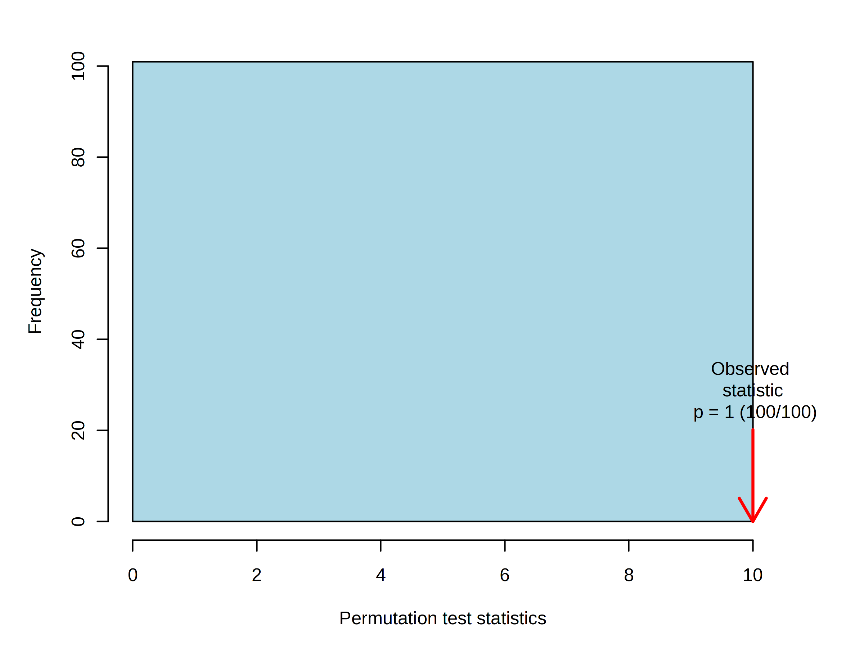


**Supplementary Figure S6:** Permutation testing (n = 100 permutations) showing no statistically significant class separation, supporting the exploratory nature of the PLS-DA analysis.
